# The VITACORA- 19 and PsAID questionnaires are equally valid for assessing the impact of psoriatic arthritis on patients' quality of life

**DOI:** 10.1007/s10067-025-07446-4

**Published:** 2025-04-21

**Authors:** Ignacio Braña, Marta Loredo, Juan Carlos Torre-Alonso, Rubén Queiro

**Affiliations:** 1 Rheumatology & ISPA Translational Immunology Division, Hospital Universitario Central de Asturias, Avenida de Roma, S/N 33011 Oviedo, Spain; 2 Department of Medicine, Facultad de Medicina de La Universidad de Oviedo, Oviedo, Spain; 3 Instituto de Investigación Sanitaria del Principado de Asturias (ISPA), Oviedo, Spain

**Keywords:** Disease impact, PsAID, Psoriatic arthritis, Quality of life, VITACORA- 19

## Abstract

**Background and aims:**

Health-related quality of life (HRQoL) is an often-underestimated aspect in psoriatic arthritis (PsA). We aimed to compare the VITACORA-19 and PsAID (Psoriatic Arthritis Impact of Disease) questionnaires to assess QoL in routine PsA management.

**Methods:**

Cross-sectional analysis of a randomly selected PsA population. Disease activity was estimated using the DAPSA (Disease Activity score for PsA) index and HRQoL using the VITACORA-19 and PsAID. The construct validity of VITACORA-19 was analysed (Pearson correlation and ROC curves).

**Results:**

Forty-five patients were included, 24 men and 21 women, mean age 55 ± 13 years, mean disease duration 8.2 ± 6.1 years. Most patients showed adequate disease control, median DAPSA 11.3 (IQR: 8.0–19.3), median PsAID 2.7 (IQR: 1.1–5.0). VITACORA-19 scores ranged from 6 to 94. The correlation between VITACORA-19 and PsAID was high, r: -0.7 (95%CI: -0.84 to -0.46), *p* < 0.0001. A VITACORA-19 score in the range of 6–29 corresponded to high DAPSA disease activity, a range of 30–44 corresponded to moderate DAPSA activity and a range of 45–95 corresponded to low DAPSA activity. The cut-off for an acceptable symptomatic state (PsAID < 4) corresponded to a VITACORA-19 score ≥ 66 with an area under the ROC curve of 0.85 (95%CI: 0.71–0.98).

**Conclusions:**

This is the first study comparing the VITACORA-19 and PsAID questionnaires. Either of the two questionnaires could be used to assess HRQoL in PsA. For the first time, VITACORA-19 thresholds are defined that identify the different DAPSA activity categories. A VITACORA-19 score ≥ 66 could be an appropriate treatment target.

**Table:** 

**Key Points**
• *Health-related quality of life (HRQoL) is an often-underestimated aspect in psoriatic arthritis (PsA)*. • *The PsAID and VITACORA-19 questionnaires offer similar performance for assessing HRQoL in PsA*. • *A VITACORA-19 ≥ 66 identifies the low disease impact state according to the PsAID*. • *VITACORA-19 thresholds identifying DAPSA categories are reported*.

## Introduction

Psoriatic arthritis (PsA) is a multi-domain disease in which musculoskeletal and cutaneous manifestations converge to varying degrees. All these aspects need to be considered when addressing disease activity and the overall impact of the disease on the lives of people with PsA [1–5]. However, instruments designed to assess health-related quality of life (HRQoL) in PsA are rarely used in routine clinical practice.

The PsA Impact of Disease (PsAID) questionnaire is the gold standard for estimating the impact of PsA on QoL [6], but according to recent interpretations it also behaves as a multipurpose tool [7, 8]. At OMERACT 2018, PsAID was provisionally endorsed as a core outcome measure for disease-specific HRQoL in PsA clinical trials. [9].

The VITACORA-19, another HRQoL instrument, has shown adequate validity, reliability and sensitivity to change in Spanish patients with PsA [10]. It has also shown good psychometric properties in Turkish PsA population [11]. In addition, VITACORA-19 is an instrument with items not covered by the PsAID and could therefore offer a broader view of very relevant nuances of HRQoL that are not only related to pain but also to physical function, work capacity or social functioning. However, almost nothing is known about how PsAID and VITACORA-19 perform when confronted with each other in daily clinical practice. Therefore, we aimed to compare the performance of the two instruments in real life.

## Patients and methods

### Design and study population

Cross-sectional study of forty-five patients that were randomly and consecutively selected from a database of patients with PsA who met the CASPAR criteria [12]. These patients regularly attended a consultation dedicated to the management of this pathology in a tertiary-care centre in north-western Spain. The recruitment period ran from October to December 2022. To avoid overestimating the responses to the questionnaires tested in the study, patients with a diagnosis of fibromyalgia or major psychiatric disorders (except for depression) were excluded. Patients were also excluded if they were on sick leave or in litigation for disability due to illness. Patients who did not have sufficient reading comprehension to complete the questionnaire were also excluded. This study was conducted in full conformance with the Spanish SAS Order/3470/2009 of the Ministry of Health and Social Policy, local laws and regulations, and the ethical principles laid down in the Declaration of Helsinki. Patients were selected from the "Spanish Registry of Patients with Psoriatic Arthritis Treated with Biologic and Small Molecule Therapies", approved by the Ethics Committee of the Principality of Asturias with Endorsement #248/19.

### Study variables

Age, sex, weight, BMI, disease duration, type of psoriasis, nail involvement, number of painful joints, number of swollen joints, presence of dactylitis and enthesitis, type of dominant joint involvement in the last 3 years of follow-up, cardiometabolic comorbidities and treatments received at enrolment were collected. The Disease Activity score for PsA (DAPSA) was used to assess disease activity [13]. Depending on the result, disease activity can be classified into four groups: remission (0–4 points), low disease activity (5–14 points), moderate disease activity (15–27 points) and high disease activity (> 27 points).

### Quality of life assessment

To assess the impact of the disease on patients' quality of life, we used the PsAID questionnaire [6]. PsAID-12 includes the following items: pain, fatigue, skin problems, work and leisure, disability in daily activities, feeling of discomfort and irritation, sleep disturbance, coping with the disease, anxiety and uncertainty, embarrassment, social participation and depression. Each item has its own weighting. The global score ranges from 0 (best) to 10 (worst). A PsAID score below 4 is considered an acceptable symptomatic state for patients [6]. We also used the VITACORA-19 questionnaire. VITACORA-19 was developed through literature review, expert consultations, and focus groups with PsA patients to ensure comprehensive coverage of relevant HRQoL aspects. In the original validation study, significant differences (*p* < 0.001) were observed between healthy controls, non-PsA patients, and PsA patients, confirming the instrument's ability to distinguish groups [10]. VITACORA-19 includes the following 19 items: mobility (item 1), ability to recover after exertion (item 2), difficulty in changing position in bed (item 3), loss of strength (item 4), lack/loss of motivation (item 5), sadness and depression due to exhaustion (item 6), mood affected by pain (item 7), fear of being in pain (item 8), fear of being dependent on others (item 9), feeling of hopelessness due to pain (item 10), reduction of social activities (item 11), reduced performance at work or at home (item 12), fear of losing job (item 13), feelings of rejection due to skin appearance (item 14), difficulty in performing tasks with hands (item 15), sleep disturbance because of pain (item 16), importance of pain in the overall context of the disease (item 17), importance of joint swelling in the overall context of the disease (item 18), and concern about the future evolution of the disease (item 19). For each of the 19 items, this instrument allows 5 Likert-like response choices from 'always' to 'never', and the time period referred to is the previous week [10]. Score for the overall questionnaire is obtained by adding the responses to the corresponding items, with subsequent standardization to a scale ranging from 0 (worst HRQoL) to 100 (best HRQoL). Standardization is obtained as follows: (actual score − minimum score) ÷ (maximum score − minimum score) × 100.

### Statistical analysis

As for the statistical analysis, descriptive statistics were first carried out with measures of central tendency and dispersion. Thus, for normally distributed variables, the mean was given with its standard deviation, while for non-normally distributed variables, the median was given with its interquartile range (IQR). Qualitative variables are expressed in absolute and relative percentage terms. The convergent validity of the VITACORA-19 questionnaire was analysed by Pearson correlations between its scores and those of the PsAID. The discriminant validity of both questionnaires was established by comparing the scores obtained in each questionnaire with the different cut-off points of the DAPSA, which is currently one of the most recommended tools for measuring disease activity in PsA in clinical practice [14]. For the latter, probabilistic graphs were constructed between the VITACORA-19 values, the PsAID values, and the different DAPSA categories, thus establishing the different score ranges that identify each category. Finally, the area under the ROC curve was used to determine the best cut-off point (Youden index) of VITACORA-19 to define an acceptable symptomatic state (PsAID < 4). As this was just an exploratory observational study, no a priori sample size was estimated. The level of statistical significance was set at *p* < 0.05. Statistical analysis was conducted using the R software (version 4.3.1, "Beagle Scouts"; R Project for Statistical Computing).

## Results

### Summary of study population

The mean age of the patients was 55.1 ± 13.0 years, with a mean disease duration of 8.2 ± 6.1 years. Of the patients, 55.8% (n: 24) were male and 44.2% (n: 21) were female. Fifty-eight percent of the patients were being treated with biologic therapies (mostly anti-TNFα), while 42% were being treated with conventional synthetic DMARDs (usually MTX) at the time of enrolment. In terms of outcome variables, most patients had their joint disease under control with a median DAPSA of 11.3 (IQR: 8.0–19.3). The impact on QoL was also low in most patients, with a median PsAID of 2.7 (IQR: 1.1–5.0). Median VITACORA-19 was 64.5 (IQR: 57.9–73). The main characteristics of the study population are summarised in Table 1.

**Table 1:** Study population characteristics

Study variables	N: 45
Age, years, mean (SD)	55.1 (13.0)
Men, n (%)	24 (53.3)
Women, n (%)	21 (46.7)
Disease duration, years, mean (SD)	8.2 (6.1)
Psoriasis family history, n (%)	22 (48.9)
PsA family history, n (%)	6 (13.3)
Weight, kilos, mean (SD)	84.7 (15.1)
BMI, mean (SD)	28.6 (6.2)
Plaque psoriasis, n (%)	38 (84.4)
Nail psoriasis, n (%)	25 (55.6)
≥ 3 body surface area, n (%)	18 (40)
Joint patterns:	
- Axial, n (%)	3 (6.7)
- Oligoarthritis, n (%)	24 (53.3)
- Polyarthritis, n (%)	18 (40.0)
Dactylitis, n (%)	16 (35.6)
Enthesitis, n (%)	20 (44.4)
Obesity, n (%)	12 (26.7)
Smokers, n (%)	10 (22.2)
Hypertension, n (%)	14 (31.1)
Diabetes, n (%)	4 (8.9)
Dyslipidemia, n (%)	15 (33.3)
Non-alcoholic fatty liver, n (%)	8 (17.8)
Cardiovascular disease, n (%)	3 (6.7)
Cancer history, n (%)	2 (4.4)
Depression, n (%)	9 (20.0)
TJC, median (IQR)*	0 (0–1.5)
SJC, median (IQR)*	0 (0–1.0)
HAQ, median (IQR)*	0.6 (0.0–1.6)
DAPSA, median (IQR)*	11.3 (8.0–19.3)
VITACORA-19, median (IQR)*	64.5 (57.9–73)
PsAID, median (IQR)*	2.7 (1.1–5.0)

*N,n* number; *SD* standard deviation; *PsA* psoriatic arthritis; *BMI* body mass index; *TJC* tender joint count; *IQR* interquartile range; *SJC* swollen joint count; *HAQ* health assessment questionnaire; *DAPSA* disease activity score for psoriatic arthritis; *PsAID* psoriatic arthritis impact of disease. *At the time of recruitment

### Convergent and discriminant validity of the VITACORA- 19

**Fig. 1:**
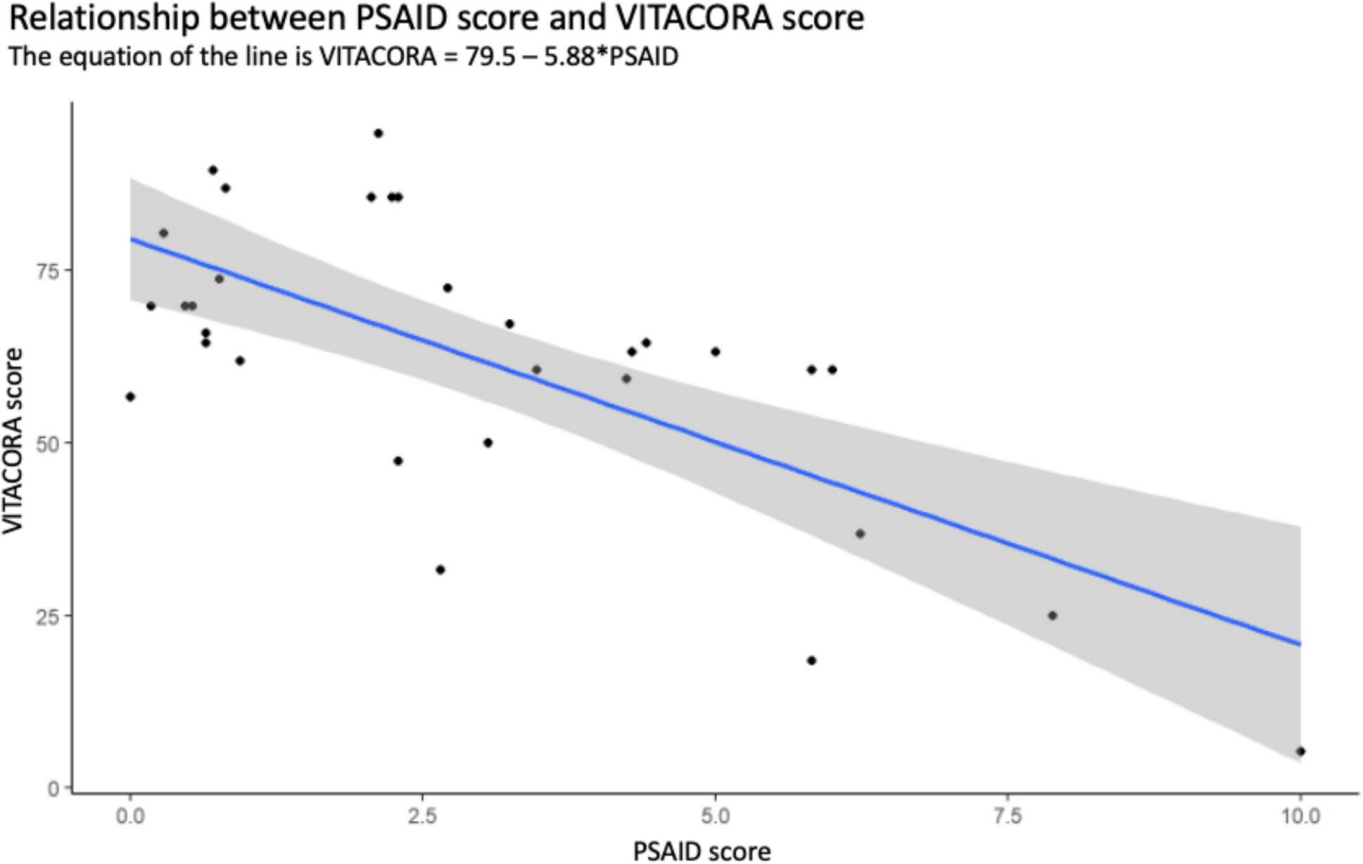
Linear correlation between VITACORA-19 and PsAID. A high correlation was found between the scores of the two questionnaires. The slope of this correlation is negative because the values go in opposite directions, i.e. in VITACORA-19 the higher the value the better the quality of life, while in PsAID the opposite is true, PsAID: psoriatic arthritis impact of disease

**Fig. 2:**
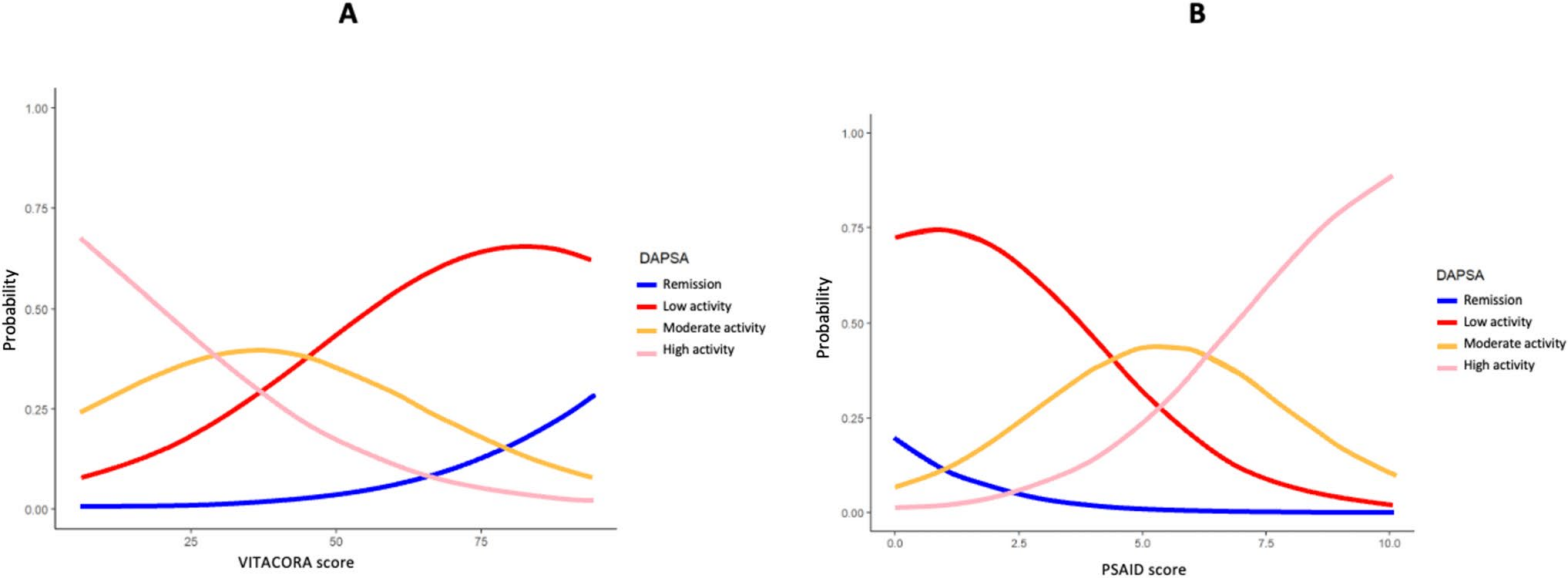
Probabilistic graphs between VITACORA-19, PsAID and DAPSA categories, The graphs represent the probability that a given VITACORA-19 (A) or PsAID (B) value identifies a given PsA activity category according to the DAPSA index. See the text for further explanation, PsAID: psoriatic arthritis impact of disease; DAPSA: disease activity score for psoriatic arthritis

**Fig. 3:**
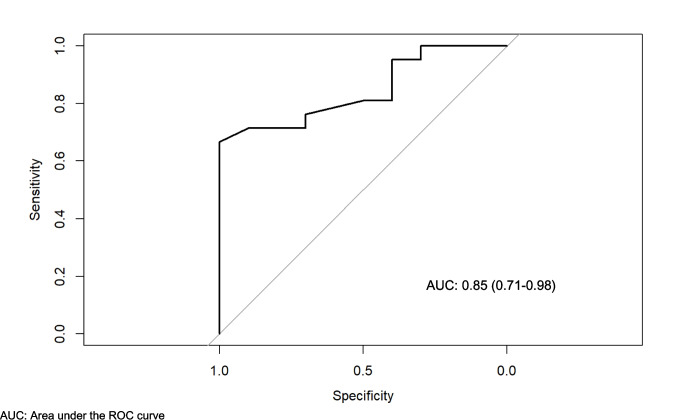
VITACORA-19 value to determine an acceptable symptomatic state according to the PsAID threshold, The area under the ROC curve is plotted. According to the Youden index, a VITACORA-19 ≥ 66 has adequate sensitivity and specificity to detect the PsAID value that determines an acceptable symptomatic state for the patient or low-impact disease (PsAID < 4), ROC: receiver operating characteristic; PsAID: psoriatic arthritis impact of disease

A high correlation was found between VITACORA-19 and PsAID scores (Fig. 1). The correlation between the two was—0.7 (95% CI: −0.84, −0.46). The equation resulting from this correlation was = 79.5—5.88*PSAID. The 95% CI for the slope of the line was between −8.17 and −3.58 (*p* < 0.00001). The discriminant validity of the two QoL questionnaires was then analysed. For each VITACORA-19 score and each PsAID score, the probability that the patient was in one of the 4 DAPSA categories was estimated. The range of VITACORA-19 scores was from 6 to 94. Thus, for a patient with a VITACORA-19 value of 6 (i.e. very poor HRQoL), the calculated probability of that patient having a DAPSA > 27 (high disease activity) was almost 70%, while the probability of that same patient being in remission DAPSA (< 4) was reduced to 0.4%. In contrast, a patient with a VITACORA-19 score of 94 had only a 2% chance of being in high DAPSA, while the chance of being in low-remission DAPSA was 90% (Fig. 2). PsAID scores ranged from 0 to 10. A patient with a PsAID of 0 (i.e. best possible QoL) had a 92% chance of being in DAPSA remission, while the chance of being in a highly active DAPSA category was only 1.2%. A patient with PsAID 10 (worst possible QoL) had an 88% chance of being in a high-activity DAPSA category and only a 0.004% chance of being in remission (Fig. 2). The final resulting model classified subjects with a VITACORA-19 score between 6 and 29 as high DAPSA activity, subjects in the range 30 to 44 as moderate activity, and 45 to 95 as low activity. For PsAID, the model classified subjects with a PsAID score between 7 and 10 as high DAPSA activity, subjects with a PsAID score between 5 and 6 as moderate DAPSA activity, and subjects with a PsAID score of 4 or less as low DAPSA activity. Finally, a VITACORA-19 ≥ 66 determined a PsAID < 4 (acceptable symptomatic status or low impact disease) with an area under the ROC curve of 0.85 (0.71–0.98) as shown in Fig. 3.

## Discussion

In this exploratory study, the comparative scores of the PsAID and the VITACORA showed a high correlation, giving the VITACORA-19 adequate construct validity for estimating QoL compared with the standard instrument for this purpose in PsA. Both instruments showed good discriminant validity in identifying the different categories of the DAPSA, one of the standard tools recommended by EULAR for estimating inflammatory disease activity [14]. Finally, a VITACORA-19 value ≥ 66 discriminated with high accuracy those patients with PsAID < 4, the threshold accepted as low disease impact. Therefore, our results support the use of both questionnaires to estimate the impact on QoL in the routine care of these patients.

Our study is the first to compare the PsAID and the VITACORA-19, two of the HRQoL questionnaires designed specifically for PsA [6, 10]. Although different tools can adequately assess HRQoL in PsA, few of them are used in clinical routine, and currently the only one of these instruments that has been endorsed by OMERACT-GRAPPA is the PsAID [9].

PsAID was translated and evaluated in several languages during the development phase and appeared a good candidate based on strong evidence for content validity and moderate evidence for good test–retest reliability and for good construct validity (external relationships) of the 12-item version (PsAID-12). Similar findings existed for PsAID-9 except that evidence for construct validity was limited. Floor/ceiling effects of PsAID were < 1%, and values for low disease impact were provided [6, 15]. VITACORA-19 was evaluated in Spanish (origin) and in Turkish resulting in moderate evidence for test–retest reliability (ICC = 0.94), content validity and construct validity (external relationships) as well as limited evidence for uni-dimensionality, internal consistency (Cronbach α = 0.95) and good structural validity [10, 11, 15]. Floor/ceiling effects were < 1% and MCID was defined [10]. However, no formal English translation or cross-cultural validation are still available. In addition to the above, we have demonstrated adequate construct and discriminant validity for the VITACORA-19 questionnaire in relation to two standard measures in PsA, such as the PsAID and the DAPSA, which allows us to recommend its use in clinical routine.

The main limitations of our study are, among others, the relatively small number of patients included, the low representativeness of patients in the highest DAPSA categories, and the fact that, as noted above, there is no cross-cultural validation of the VITACORA-19 which restricts its use to the original design setting. Another weakness is that the assessment of psoriasis was made by evaluating the number of patients with 3 or more body areas affected by psoriasis, while a more common tool such as the PASI was not used. In any case, given its adequate performance relative to the PsAID and DAPSA, we believe that the VITACORA-19 captures important aspects of the disease, which can certainly go beyond the simple estimation of QoL. As a novelty, and for the first time, a cut-off value (VITACORA-19 ≥ 66) has been obtained that corresponds reasonably well with the low disease impact state characterised by the PsAID, so that this VITACORA-19 threshold can be used as a therapeutic target in the management of PsA. We have also identified the VITACORA-19 thresholds that identify the different categories of disease activity according to DAPSA. This information is undoubtedly of interest to practitioners, as QoL is an often-neglected aspect of PsA management with important implications for selecting the right treatment for each patient [16–18].

In conclusion, the VITACORA-19 has some psychometric properties similar to those of the PsAID. Although its use is limited to the Spanish and Turkish settings, our results may encourage other non-Spanish-speaking authors to try to validate it in other settings and cultures.

## Data Availability

The data on which this study is based are available to third parties upon reasonable request.
